# Coexistence of Gynecological Pathologies in Women With Cervical and Endometrial Polyps

**DOI:** 10.7759/cureus.77015

**Published:** 2025-01-06

**Authors:** Aikaterini Sidera, Michail Matalliotakis, Ioannis Tsakiridis, Charoula Matalliotaki, Konstantinos Krithinakis, Themistoklis Dagklis, Apostolos M Mamopoulos, Ioannis A Kalogiannidis

**Affiliations:** 1 Third Department of Obstetrics and Gynaecology, Aristotle University of Thessaloniki, Thessaloniki, GRC; 2 Department of Obstetrics and Gynaecology, Venizeleio General Hospital, Heraklion, GRC

**Keywords:** benign, cervical polyp, endometrial polyp, gynecological pathology, malignant

## Abstract

Introduction: Cervical polyps (CPs) and endometrial polyps (EPs) are common gynecological conditions worldwide. Understanding the distribution of benign and malignant pathologies in women with these polyps is clinically significant. The coexistence of these conditions underscores their complexity, requiring an inclusive and detailed approach to diagnosis and management. This study aimed to investigate the distribution of coexisting pathologies in women with CPs and EPs.

Methods: This retrospective study analyzed records from 2000 to 2020 at the Department of Obstetrics and Gynecology at Venizeleio General Hospital of Heraklion, Crete, and the Third Department of Obstetrics and Gynecology, School of Medicine, Faculty of Health Sciences, Aristotle University of Thessaloniki, Greece.

Results: From a total sample of 3,490 women, 772 women were diagnosed with one or more polyps; 343 (9.9%) had CPs, and 488 (14%) had EPs. Benign pathologies, such as cervicitis, adenomyosis, paraovarian cysts, and fibroids, were strongly associated with the presence of CPs. Regarding EPs, a positive statistical relationship was present between them and serous cystadenomas (p=0.005), cervicitis (p<0.001), adenomyosis (p=0.001), paraovarian cysts, and more than five fibroids (p<0.001). Endometrial polyps were strongly (p<0.001) correlated to endometrial hyperplasia with or without atypia. The occurrence of EPs and CPs appeared to decrease with age. Further analyses revealed a strong positive correlation between EPs and endometrial cancer (p=0.006). Endometrial polyps were strongly (p<0.001) correlated to endometrial hyperplasia with or without atypia. The occurrence of EPs and CPs appeared to decrease with age. Further analyses revealed a strong positive correlation between EPs and endometrial cancer (p=0.006).

Conclusions: There appears to be a correlation between EPs/CPs and other gynecological pathologies. Comprehensive evaluation and further longitudinal studies are therefore crucial for patients with polyps, as they may be at higher risk for coexisting benign and potentially malignant conditions.

## Introduction

Both endometrial polyps (EPs) and cervical polyps (CPs) are common gynecological conditions affecting women worldwide. These benign growths arise from the epithelial lining of the genital tract and are often asymptomatic, frequently being discovered incidentally during routine examinations; their clinical significance lies in their potential coexistence with various benign and malignant gynecological pathologies [1-3].

The prevalence of EPs is reported to be 8%-50%, while CPs affect up to 10% of women [4-6]. Clinically, genital tract polyps may manifest with symptoms such as menorrhagia, intermenstrual bleeding, infertility, and postmenopausal bleeding [3,7]. Understanding the distribution and associated pathologies of these polyps may enhance clinical outcomes and update treatment strategies [1,8].

The pathogenesis of CPs and EPs may be influenced by a complex interplay of genetic, hormonal, and environmental factors; estrogen receptors (ERs) in the endometrium play a critical role, with abnormal ER expression linked to conditions such as endometriosis, endometrial hyperplasia, and endometrial cancer [9,10]. Estrogen, particularly when unopposed by progesterone, promotes endometrial cell proliferation, contributing to polyp formation, especially in cases of hyperestrogenism commonly seen in obesity and polycystic ovary syndrome [5,11]. Tamoxifen, a medication with estrogen-like effects, has also been associated with EPs due to its role in promoting cell survival and inhibiting apoptosis, with its specific impact varying based on dosage and duration [12,13]. Additionally, genetic predisposition and mutations that affect cell growth and apoptosis, particularly through increased Bcl-2 expression, play a key role in EP development by inhibiting apoptosis and preventing normal tissue shedding [14,15]. Chronic inflammation, characterized by elevated cytokines and matrix metalloproteinases, further disrupts endometrial architecture, promoting polyp formation [16].

Histological examination of polyps reveals distinct features that aid in their diagnosis and differentiation from other gynecological conditions [17]. These features are crucial for differentiating polyps from endometrial hyperplasia or malignancy. Cervical polyps, on the other hand, are often composed of fibrous stroma covered by endocervical or ectocervical epithelium; they may appear as pedunculated or sessile growths and can vary in color from cherry-red to pale grey [18].

The primary purpose of this study was to investigate the coexistence of benign and malignant gynecological pathologies in women diagnosed with EPs and CPs, aiming to identify potential associations between these conditions.

## Materials and methods

This retrospective study analyzed records from 2000 to 2020 at the Department of Obstetrics and Gynecology at Venizeleio General Hospital of Heraklion, Crete, and the Third Department of Obstetrics and Gynecology, School of Medicine, Faculty of Health Sciences, Aristotle University of Thessaloniki, Greece. Out of 3,490 women with various gynecological conditions who underwent surgical interventions, i.e., total hysterectomy with or without unilateral or bilateral salpingo-oophorectomy, hysteroscopy, and/or dilation and curettage (D&C), we retrospectively studied cases that were diagnosed with histologically confirmed CPs and EPs. Reported gynecological comorbidities included cervicitis, endometriosis, and adenomyosis, as well as masses such as CPs and EPs, cysts, cystadenomas, teratomas, endometriomas, and fibroids, in addition to ovarian, cervical, and endometrial malignancies. Cases with incomplete medical records or lacking histological results were excluded. The research protocol received approval from the ethics committee of both departments (protocol numbers: 124/17/2019 and 94/23-4-20).

Statistical analyses were performed using IBM SPSS software version 25 (IBM Corp., Armonk, NY). Descriptive statistics were used to calculate the mean, average, and standard deviation of all data. Values were considered statistically significant at p<0.05. The results are reported as mean ± SD or as percentages, where appropriate. Graphs were generated using the Prism software (GraphPad Software, La Jolla, CA).

## Results

**Figure 1:**
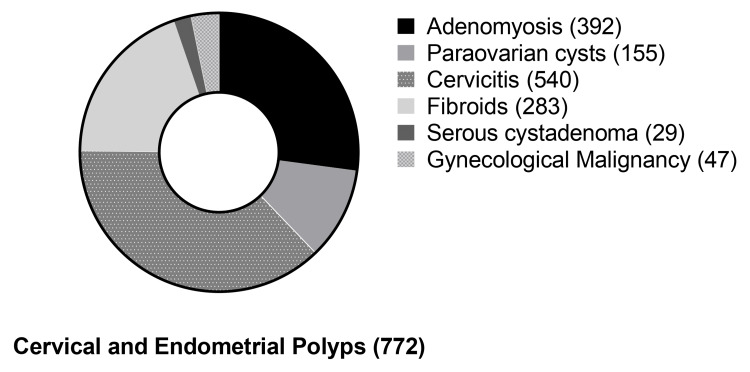
Distribution of the most common benign and malignant gynecological pathologies among women with cervical and endometrial polyps

Over a two-decade period, out of 3,490 women, 343 (9.9%) were diagnosed with histologically confirmed CPs, 488 (14%) with EPs, while 61 women had both EPs and CPs, with a mean age of 50.90±10.17 years (range 21-82 years), 59.08±10.78 years (range 25-84 years), and 53.49±10.23 years (range 25-81 years), respectively (Table 1). Polyps coexisted with a number of conditions in our sample (Figure 1, Table 2).

**Table 1:** Prevalence of polyps and their distribution according to age

Type of polyp	Number of patients	Proportion (%)	Mean age (years)
Cervical polyps	343	9.9%	50.9 ± 10.1
Endometrial polyps	488	14%	59.0 ± 10.7
Both types	61	1.75%	53.4 ± 10.2

**Table 2:** Distribution of the most common gynecological pathologies among cervical and endometrial polyps and the total sample p-values considered statistically significant at <0.05. df=1; * denotes negative correlations

	Cervical polyps (n=343)	Endometrial polyps (n=488)	Total (n=3490)
Cervicitis	82.3%	62.3%	46.0%
	p<0.001	p<0.001	
Morgagni cysts	5.5%	6.4%	4.9%
	p=0.56	p=0.10	
Paraovarian cyst	20.0%	21.6%	14.4%
	p=0.002	p<0.001	
Walthard cell nests (fallopian tube)	2%	1.6%	1.4%
	p=0.33	p=0.68	
Serous cystadenoma	12.5%	15.4%	10.5%
	p=0.21	p<0.001	
Mucinous cystadenoma	3.2%	2.5%	3.6%
	p=0.68	p=0.16	
Mature teratoma	1.7%	2.9%	7.7%
	p<0.001*	p<0.001*	
Immature teratoma	0.3%	0.0%	0.3%
	p=0.90	p=0.23	
Thecoma	0.0%	0.6%	0.6%
	p=0.12	p=0.96	
Struma ovarii	0.3%	0.0%	0.3%
	p=0.86	p=0.16	
Brenner cyst	0.3%	0.4%	0.6%
	p=0.40	p=0.50	
Ovarian fibroma	3.2%	4.1%	2.9%
	p=0.70	p=0.08	
Endometrioma	6.4%	3.5%	13.7%
	p<0.001*	p<0.001*	
Ovarian cancer	0.9%	1.0%	1.5%
	p=0.28	p=0.31	
Borderline ovarian tumor	0.6%	0.8%	0.7%
	p=0.71	p=0.83	
Cervical cancer	4.9%	5.1%	4.2%
	p=0.50	p=0.30	
Endometrial cancer	4.3%	8.2%	5.6%
	p=0.30	p=0.006	
Endometriosis	5.8%	4.5%	4.3%
	p=0.13	p=0.78	
Endometritis	0.3%	0.0%	0.2%
	p=0.80	p=0.25	
Endometrial hyperplasia without atypia	4.1%	6.6%	3.5%
	p=0.54	p<0.001	
Endometrial hyperplasia with atypia	2.6%	4.7%	2.0%
	p=0.34	p<0.001	
Fibroids	44.6%	46.3%	37.4%
	p=0.003	p<0.001	
Adenomyosis	42.7%	55.9%	34.3%
	p=0.001	p<0.001	
Malignancy	5.8%	6.4%	6.5%
	p=0.003*	p=0.73	

Pearson’s analysis of the women with CPs showed a positive correlation coefficient with total abdominal hysterectomy with both adnexa (p<0.001), but not with one or no adnexa. Similarly, there was a positive link between CPs and serous cystadenoma on the right ovary (p=0.010), but not on the left (p=0.27) or both (p=0.35). A statistical correlation was also present with cervicitis (p<0.001), adenomyosis (p=0.001), paraovarian cysts (p=0.002), and the presence of more than five fibroids (p<0.001). Cervical polyps were strongly associated with the co-existence of other comorbidities, with p=0.002 for up to three comorbidities and p<0.001 for up to five or more than five comorbidities. A weak correlation was noticed with the presence of EPs (p=0.033).

Cervical polyps' occurrence appears to decrease with age (p<0.001), and the presence of certain masses, namely mature teratomas and endometriomas, has a strong correlation for the right ovary (p<0.001) and weak for the left (p=0.043 and p=0.027 respectively). 

An association between CPs and the presence of a malignant gynecological condition was revealed (p=0.05), albeit with no connection to a specific type of cancer.

Regarding EPs, Pearson’s analysis showed a positive correlation coefficient with total abdominal hysterectomy with both adnexa (p<0.001) and no adnexa (p=0.001), but not with one adnexa. Similarly, there was a positive correlation between EPs and serous cystadenoma on both ovaries (p=0.005), but not on the right (p=0.08) or left (p=0.05). Similar to cervical polyps, a statistical association was also present between endometrial polyps and cervicitis (p<0.001), adenomyosis (p=0.001), paraovarian cysts (p<0.001), and the presence of more than five fibroids (p<0.001). Endometrial polyps were strongly (p<0.001) correlated with endometrial hyperplasia with or without atypia, as well as the co-existence of up to five or more than five other comorbidities. endometrial polyps’ occurrence appears to decrease with age (p<0.001), and the presence of certain masses, namely endometriomas on the right or left ovary (p<0.001) and mature teratomas with a strong correlation (p<0.001) for the right ovary and weak for the left (p=0.038). 

Concerning the existence of gynecological malignancies in general and EPs, there was no link. However, there was a strong correlation between EPs and endometrial cancer stage 1 (p=0.005), as well as endometrioid endometrial cancer (p=0.001).

## Discussion

The authors aimed to provide an extensive analysis of the prevalence, clinical correlations, and epidemiological characteristics of cervical and EPs over a long period of time. The prevalence of both EPs aligns with figures reported in the literature ranging from 7.8% to 50%, being more common in women between the ages of 30 and 50 [4,9], as well as the prevalence of cervical polyps reported in up to 10% of women [6].

The pathogenesis of CPs and EPs is influenced by a complex interplay of genetic, hormonal, and environmental factors. Estrogen receptors in the endometrium play a critical role, with abnormal ER expression linked to conditions such as endometriosis, endometrial hyperplasia, and endometrial cancer [10]. Estrogen, particularly when unopposed by progesterone, promotes endometrial cell proliferation, contributing to polyp formation, especially in cases of hyperestrogenism commonly seen in obesity and polycystic ovary syndrome [5,11]. Tamoxifen, a medication with estrogen-like effects, has also been associated with EPs due to its role in promoting cell survival and inhibiting apoptosis, with its specific impact varying based on dosage and duration [12,13]. Additionally, genetic predisposition and mutations that affect cell growth and apoptosis, particularly through increased Bcl-2 expression, play a key role in EP development by inhibiting apoptosis and preventing normal tissue shedding [14,15]. Chronic inflammation, characterized by elevated cytokines and matrix metalloproteinases, further disrupts endometrial architecture, promoting polyp formation [16].

Histological examination of polyps reveals distinct features that aid in their diagnosis and differentiation from other gynecological conditions. Endometrial polyps exhibit hyperplastic endometrial glands surrounded by a stroma rich in blood vessels. The irregularity of glandular proliferation and the presence of fibrous tissue are characteristic of EPs [17]. These features are crucial for differentiating polyps from endometrial hyperplasia or malignancy. Endometrial polyps often present with symptoms such as menorrhagia, intermenstrual bleeding, and, occasionally, infertility [7].

Cervical polyps, on the other hand, are often composed of fibrous stroma covered by endocervical or ectocervical epithelium. They may appear as pedunculated or sessile growths and can vary in color from cherry-red to pale grey [18]. While generally asymptomatic, cervical polyps can cause symptoms such as postcoital bleeding, abnormal discharge, and, in some cases, pain [3].

Previous research has explored risk factors associated with endometrial polyps in depth. Savelli et al. aimed to elucidate the prevalence of benign, hyperplastic, and malignant EPs and to identify clinical predictors of histopathologic outcomes; after analyzing 509 patients who underwent hysteroscopic removal of EPs, it was found that the majority of polyps (70.3%) were benign. The study highlighted that age, menopausal status, and hypertension were significant risk factors associated with more severe histopathologic findings, including atypical hyperplastic and cancerous polyps [19].

Our data showed significant associations between CPs and EPs and various benign gynecological conditions. Moreover, the coexistence of multiple gynecological diseases may suggest common underlying factors, possibly related to estrogenic effects [20]. In our study, there were 61 cases (2%) of patients with concurrent CPs and EPs. Goeman et al. reported that 26.7% of patients with CPs also had EPs, indicating a potential shared pathophysiological mechanism. This incidence was significantly lower (8.3%) among those on combined pill treatment [21]. Vilodre et al. found that the prevalence of EPs was significantly higher in women with a CP (26.9%) compared to those without (7.1%) [22].

Furthermore, we observed that 44.6% of women diagnosed with CPs and 46.3% of those with EPs were also found to have fibroids. Kinay et al. found that 20.1% (155 out of 772) of patients had coexisting uterine fibroids and EPs. The study identified several factors associated with this coexistence, including age, hypertension, endometrial hyperplasia, CPs, and the number of fibroids [23].

Some authors propose a shared etiological origin for EPs and endometriosis, emphasizing unopposed estrogenic stimulation. Serteva et al. identified significant coexistence of endometriosis with other uterine conditions: endometrial atrophy in 28 cases (12.5%), EPs in 23 cases (10.3%), and endometrial carcinoma in 11 cases (5.8%) [24]. In a retrospective study of 431 infertile women, a significantly higher frequency of EPs was found in those with endometriosis compared to those without. The study also demonstrated that hysteroscopic polypectomy, combined with the removal of endometriotic foci, significantly improved pregnancy outcomes for patients with polyps, regardless of the stage or location of endometriosis [25].

Indraccolo and Barbieri observed a link between adenomyosis and uterine polyps in 17.6% of the cases, highlighting the role of hyperestrogenemia in EP formation [26]. Tetikkurt et al. identified EPs in 22.3% (71 of 319) of patients with adenomyosis [27].

Furthermore, we identified a coexistence of EPs and endometrial hyperplasia with and without atypia. Bakour et al. reported that from the 248 EP histologic specimens, 85.5% were benign, with 11.3% showing hyperplasia and 3.2% associated with malignancy. The study highlighted that hyperplasia was more common in polyps than in non-polypoidal specimens [28]. This observation is consistent with findings from Savelli et al., where 25.7% of polyps exhibited hyperplastic changes, with a small percentage showing atypical hyperplasia (3.1%) or malignancy (0.8%) [19], and findings from Kelly et al., where it was noted that EPs are frequently accompanied by concurrent endometrial hyperplasia in the nonpolypoid endometrium in 52% of cases [29]. These findings underscore that while malignant transformation of EPs is relatively rare, hyperplastic changes are more prevalent and should not be overlooked.

Although most EPs are benign, their association with malignancy, particularly in postmenopausal women, underscores the importance of thorough histological evaluation. Key risk factors for identifying malignancy in EPs include menopause, abnormal uterine bleeding, obesity, polyps >1 cm, multiple polyps, nulliparity, age, and hypertension. Notably, studies have observed a rising prevalence of premalignant and malignant lesions, correlating with increases in obesity, nulliparity, and the presence of multiple polyps among patients [11,30]. Hileeto's study examined EPs and found that while 13% of all EPs were malignant, the prevalence of malignancy significantly increased with age. Specifically, only 2.5% of EPs in patients over 35 years were malignant, compared to 32% in those over 65 years. The most common malignancy associated with EPs was endometrioid adenocarcinoma [31]. In a recent meta-analysis conducted by Rijk et al., it was shown that 5.6% of cases involved endometrial cancer concurrently with atypical EPs [32]. However, other studies have suggested a higher estimate of up to 30% [33]. In our study, we found that endometrial cancer was present in 8.6% of cases where a specific histological type for the EP was not identified. In the context of CPs, malignant gynecological conditions are rare, and no strong association was found in our study [34]. A recent study by Chu et al. reported that CPs are predominantly benign, with malignancy being exceedingly rare and only a small fraction of cases exhibiting premalignant lesions [35].

With the increased utilization of transvaginal ultrasound in routine gynecological check-ups, more asymptomatic pathologies are being identified in women even in the absence of symptoms [36]. While D&C was once the standard approach for the diagnosis and treatment of EPs, hysteroscopy is now the gold standard due to its superior sensitivity and specificity [17]. Diagnosis typically involves removing the polyp through techniques such as gentle twisting or using punch biopsies. These methods allow for accurate assessment and histological examination to confirm the diagnosis [37]. Hysteroscopy allows for a thorough exploration of the cervical canal and uterine cavity, enabling precise identification of the polyp's origin (cervical or endometrial) and assessment for any accompanying endometrial pathology [7].

With regard to the limitations of this study, its retrospective design and reliance on medical records may introduce certain biases, including selection and information bias. Furthermore, the findings are based on data from two centers, so they are not generalizable for the total Greek population. On the other hand, the strong points are the large number of cases and the selection of all specimens based on histological biopsy.

## Conclusions

Both CPs and EPs are linked with various gynecological conditions and comorbidities. cervical polyps were found to have significant associations with cervicitis, adenomyosis, paraovarian cysts, and fibroids. Endometrial polyps demonstrated strong correlations with the same conditions, in addition to a notable association with endometrial hyperplasia and endometrial cancer. These findings highlight the complexity of polyps and their associated conditions, underscoring the need for a thorough and multidisciplinary approach to diagnosis and management. These findings enhance the understanding of gynecological pathology and set the stage for future research aimed at refining diagnostic criteria and developing targeted therapeutic strategies.
